# The Inhibition of miR-144-3p on Cell Proliferation and Metastasis by Targeting TOP2A in HCMV-Positive Glioblastoma Cells

**DOI:** 10.3390/molecules23123259

**Published:** 2018-12-10

**Authors:** Jingyi Song, Qingxia Ma, Ming Hu, Dongmeng Qian, Bin Wang, Ningning He

**Affiliations:** School of Basic Medical Sciences, Qingdao University, 38 Dengzhou Road, Qingdao 266021, China; 15265299578@126.com (J.S.); 18724702306@163.com (Q.M.); qdhm@qdu.edu.cn (M.H.); qiandongmeng@sina.com (D.Q.)

**Keywords:** glioma, HCMV, miR-144-3p, Top2A, proliferation

## Abstract

Glioblastoma (GBM), the most common type of primary tumor in the central nervous system, is a very aggressive brain tumor with poor prognosis and a high recurrence rate. Increasing evidence suggests that human cytomegalovirus (HCMV) infection is related to GBM and leads to GBM cell growth and metastasis. MicroRNAs are important regulators in the growth and metastasis of glioblastoma. This study aimed to demonstrate the role of miR-144-3p in HCMV-positive glioblastoma. We found that, after HCMV infection, the expression of miR-144-3p decreased, whereas the expression of TOP2A increased. Bioinformatics analyses indicated that miR-144-3p directly targets the TOP2A 3′-UTR (Untranslated Region). We discovered that the overexpression of miR-144-3p downregulated the overexpression of TOP2A and inhibited the proliferation, clone formation, and invasion of HCMV-positive glioma in vitro. Taken together, these results show that miR-144-3p inhibited growth and promoted apoptosis in glioma cells by targeting TOP2A.

## 1. Introduction

Glioblastoma (GBM) is one of the most common malignant brain tumors with high morbidity. It is characterized by its diffuse infiltration and is highly metastatic, which explains its median survival of 14–15 months and its two-year survival rate of 30% [1,2,3]. GBM has a poor prognosis and high recurrence, and it is extremely difficult to treat. Therefore, it is necessary to improve therapeutic approaches and find novel treatment strategies. It has been reported that most malignant GBM patients are infected with human cytomegalovirus (HCMV). This finding reflects that HCMV plays a vital role in glioma pathogenesis [4,5,6]. HCMV, belonging to the betaherpesvirus subfamily, is a common pathogen that encodes a set of proteins [7,8,9]. One of those proteins is the HCMV immediate early 1 (IE1) protein, which is detected in all glioblastomas and in 82% of low-grade gliomas [9,10,11]. It has been reported that IE1 siRNA likely reduces topoisomerase II alpha (TOP2A) expression [12].

TOP2A, a latent biomarker for cancer therapy, has been detected in various types of cancer, including breast cancer, hepatocellular carcinoma and glioma [13,14,15]. TOP2A is reported to be silenced by microRNAs (e.g., miR-139 and miR-548c-3p) [16,17] and plays a critical role in cell development, proliferation, apoptosis [18], cell invasion and cell migration [19,20]. The miRNAs, as tumor suppressors, play a critical role in cell processes, including development, proliferation and apoptosis. miR-144-3p belonging to the cluster miR-144/451 or miR-144 family, is related to tumorigenesis and widely used as a tumor inhibitor. Previous research has shown that its expression is significantly downregulated in osteosarcoma [21], lung cancer [22], prostate cancer [23] and glioblastoma [24,25].However, the molecular mechanism that regulates TOP2A expression is still unclear for HCMV-positive glioma.

In this study, we found that TOP2A was highly expressed in HCMV-positive glioma compared with HCMV-negative glioma. TOP2A is a direct target gene for miR-144-3p, and TOP2A promotes the proliferation and migration of glioma cells. Overexpression of miR-144-3p suppresses the growth of glioma xenografts in nude mice. These data suggest that miR-144-3p is likely to inhibit proliferation by targeting TOP2A in HCMV-positive glioblastoma.

## 2. Results

### 2.1. TOP2A Is Highly Expressed in HCMV-Positive Glioblastoma

**Figure 1:**
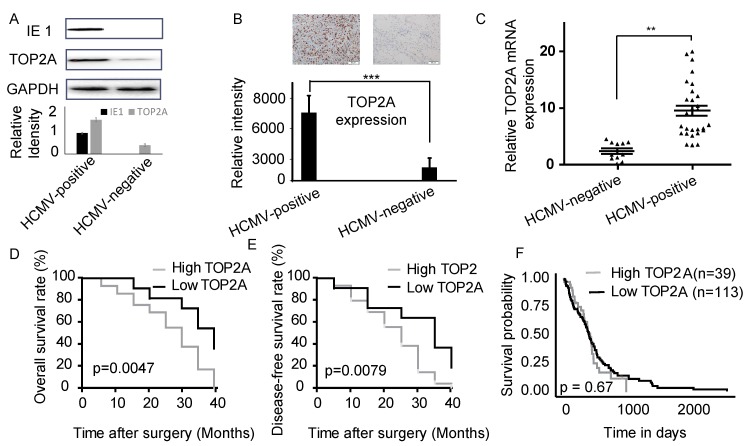
TOP2A was highly expressed in HCMV (human cytomegalovirus)-positive glioblastoma tissue. (**A**) Relative expression levels of the IE1 and TOP2A proteins were measured by western blots in HCMV-positive and HCMV-negative glioblastoma tissues. #1 sample for HCMV-positive and #10 for HCMV-negative. (**B**) The protein expression level of TOP2A was measured by immunohistochemistry in HCMV-positive and HCMV-negative glioblastoma tissues. #1 sample for HCMV-positive and #38 for HCMV-negative. (**C**) The relative mRNA expression of TOP2A was measured by qPCR in HCMV-positive (29 samples) and HCMV-negative (11 samples) glioblastoma tissues. (**D**) Patients were divided into two groups: high and low TOP2A expression, according to the mean values of the cohort. (**E**) Kaplan-Meier survival curves for glioma patients with high and low expression of TOP2A (*n* = 40). (**F**) Effects of TOP2A expression level on GBM patient survival. **: *p* < 0.01, ***: *p* < 0.001.

We measured the expression of the human cytomegalovirus immediate early protein IE1 to determine HCMV infection in the 40 glioma tissue samples by Western blots. Twenty-nine samples were HCMV-positive, and 11 samples were HCMV-negative (Figures S1 and Figure 1A). We also quantified TOP2A expression in these samples by immunohistochemistry and qPCR. The TOP2A expression level was clearly higher (the TOP2A expression more than 5 fold) in HCMV-positive tissues than in HCMV-negative tissues (Figure 1B,C and Figures S2 and S3). We also found that TOP2A expression correlated with WHO grade (*p* < 0.05) (Table 1 and Table S1). However, there was no significant correlation with patient age, gender or Karnofsky performance status. In addition, Kaplan-Meier analysis revealed that patients with high TOP2A expression (We defined the relative expression > 7 as high expression) clearly had poorer tumor-free survival and overall survival rates (Figure 1D,E). These data suggested that TOP2A was highly expressed in HCMV-positive glioma. The results from The Cancer Genome Atlas (TCGA) database demonstrated that patients with higher TOP2A expression levels consistently had poorer prognoses (Figure 1F). Although the statistical difference was not significant (*p* = 0.67), there were essential differences between the two groups.

### 2.2. TOP2A Affects HCMV-Infected Cell Viability

**Figure 2:**
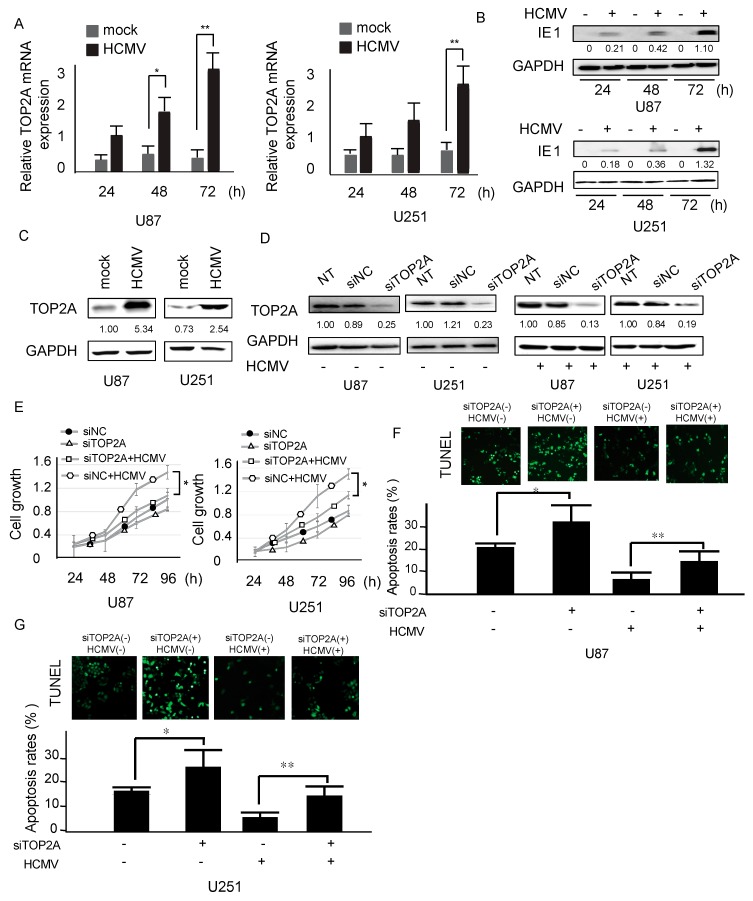
Effects of TOP2A on HCMV-infected glioma cell proliferation. (**A**) Expression of TOP2A mRNA was measured in the HCMV-positive group compared with the control group during HCMV infection. (**B**) IE1 protein expression was measured after U87 and U251 cells were infected with HCMV for 24 h, 48 h and 72 h. (**C**) TOP2A protein expression was measured after U87 and U251 cells were infected with HCMV for 72 h. (**D**) The expression of TOP2A in HCMV-positive U87 and U251 cells was measured by western blots after HCMV infection with control or TOP2A siRNA for 48 h. (**E**) Cell growth curves were measured via MTT assays (3-(4,5)-dimethylthiahiazo (-z-y1)-3,5-di-phenytetrazoliumromide). (**F**,**G**) Cell apoptosis was determined using a TUNEL assay after the cells were treated with TOP2A siRNA with or without HCMV infection. NT represent negative control (untreated cell), siNC represent the corresponding control siRNA, siTOP2A represent TOP2A-specific small interfering. For HCMV: + represent HCMV infection and − represent HCMV uninfection. For siTOP2A: + represent TOP2A siRNAs treatment; − represent control siRNAs treatment. The green fluorescence represented TUNEL staining-positive cells. *: *p* < 0.05; **: *p* < 0.01.

To explore the molecular mechanism of TOP2A in HCMV-positive glioma, we measured the transcriptional and protein expression of TOP2A in two glioma cell lines, U87 and U251, by comparing the results before and after infection with the AD169 HCMV strain. The high mRNA and protein expression (TOP2A expression level > 1) of TOP2A was verified in these two cell lines after HCMV infection (Figure 2A–C). To assess the biological role of TOP2A, TOP2A-specific small interfering RNAs (siTOP2A) or the corresponding control siRNA (siNC) was measured in HCMV-infected glioma cells, and the efficiency of TOP2A siRNAs was also tested (Figure 2D). As a result, TOP2A knockdown significantly reduced cell growth and enhanced apoptosis in glioma cells infected with HCMV (Figure 2E–G). These results indicate that TOP2A is related to antiapoptosis activity and cell proliferation in HCMV-positive glioma cells.

### 2.3. miR-144-3p Directly Targets and is Negatively Correlated with the TOP2A 3′-UTR in Glioma Cells

**Figure 3:**
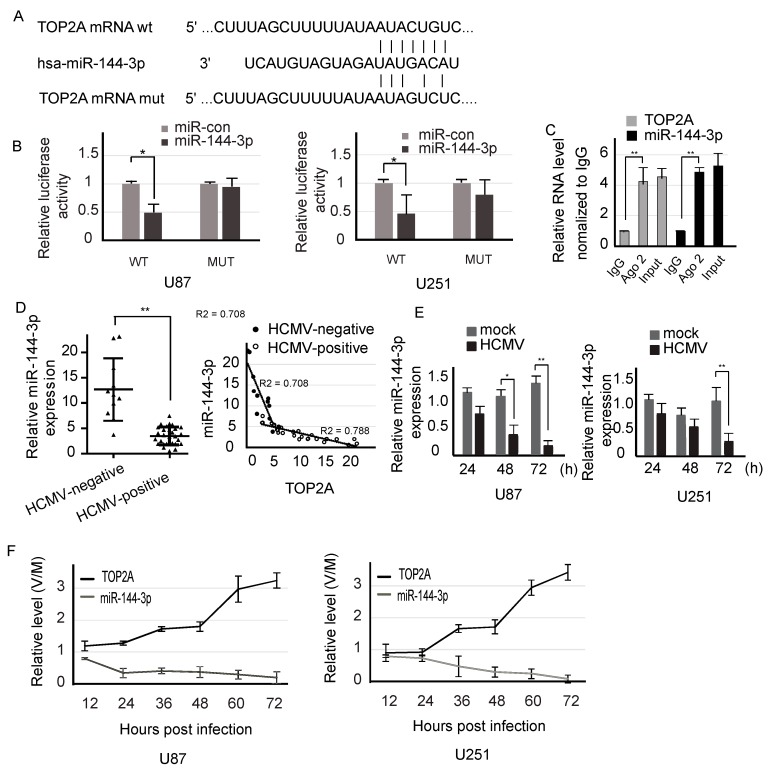
miR-144-3p directly targeted the TOP2A 3′-UTR and was negatively correlated with TOP2A expression. (**A**) The binding sites of miR-144-3p within the TOP2A mRNA 3′-UTR were predicted. The mutant type of TOP2A is also shown in the diagram. (**B**) U87 cells and U251 cells were transfected with the pMIR 3′-UTR or the mutant pMIR 3′-UTR together with miR-control or miR-144-3p. After 48 h, the relative luciferase activity was measured. (**C**) AGO2-dependent RNA immunoprecipitation (RIP) assay. IgG was used as a negative control. (**D**) The relative expression of miR-144-3p was investigated in 29 HCMV-positive tissues compared to 11 HCMV-negative tissues by qPCR. The relationship between miR-144-3p and TOP2A expression was investigated. (**E**) The relative expression of miR-144-3p in U87 and U251 glioma cell lines at different times after HCMV (V) or mock (M) infection. (**F**) miR-144-3p and TOP2A were oppositely affected by HCMV infection. *: *p* < 0.05; **: *p* < 0.01.

Using the prediction tool TargetScan for human microRNA targets [26], we found that TOP2A could potentially be targeted by miR-144-3p by directly binding to the 3′-UTR of TOP2A mRNA (Figure 3A). To confirm this hypothesis, we cloned the 3′-UTR wild-type or 3′-UTR mutant-type TOP2A into a pMIR-REPORT vector. As expected, the luciferase activity of the wild-type 3′-UTR in the cells transfected with miR-144-3p was much lower than that in cells transfected with the miR-control, while the mutant-type 3′-UTR exhibited almost no luciferase activity (Figure 3B). To further explore the relationship between endogenous miR-144-3p and TOP2A in RISC complex, we used Ago2 to explore the interaction between endogenous miRNAs and mRNAs in RISC complex. The results indicate that there is a direct interaction between endogenous miR-144-3p and TOP2A in RISC complex (Figure 3C). Hence, miR-144-3p directly targets TOP2A.

The expression of miR-144-3p was detected in glioma tissues and cell lines. The expression of miR-144-3p in the HCMV-positive samples was clearly lower than that in the HCMV-negative samples, and miR-144-3p expression was negatively correlated with TOP2A expression (Figure 3D). Consistent with the tissue results, the miR-144-3p level was lower in the HCMV-positive cells than in the HCMV-negative cells (Figure 3E). Furthermore, we measured the expression of TOP2A and miR-144-3p during HCMV infection of glioma cell lines with miR-144-3p, anti-miR-144-3p and untreated. During the time course, the expression of TOP2A increased, whereas the expression of miR-144-3p decreased (Figure 3F and Figure S4). These results indicate that miR-144-3p and TOP2A were inversely affected by HCMV infection.

### 2.4. miR-144-3p Inhibits Cell Growth, Clone Formation and Migration

**Figure 4:**
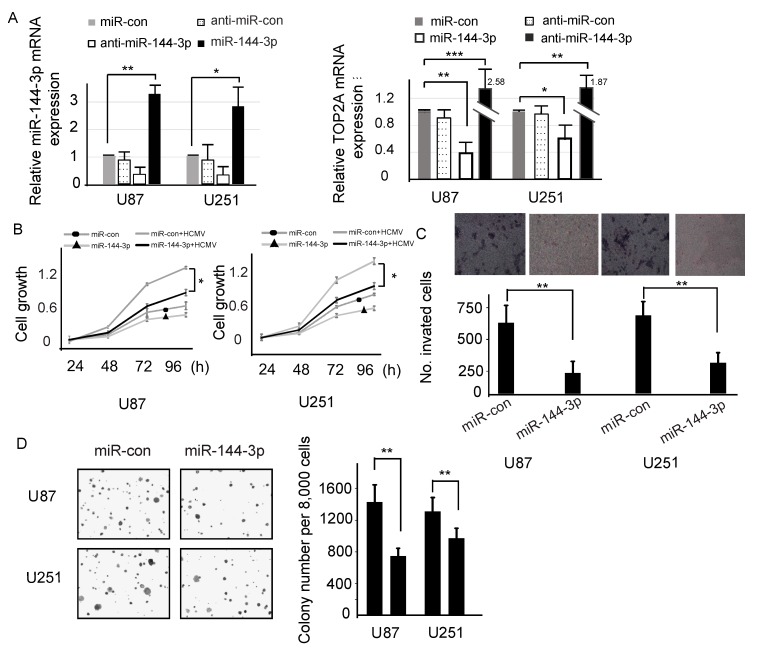
miR-144-3p inhibited the proliferation, formation and migration of HCMV-positive glioma cells. (**A**) The expression of miR-144-3p in HCMV-infected U87 cells and U251 cells was measured by qPCR after transfection with miR-controls, miR-144-3p or anti-miR-144-3p. The mRNA expression level of TOP2A decreased with the transfection of miR-144-3p in HCMV-infected U87 and U251 cells. (**B**) The effects of miR-144-3p on the cell proliferation of HCMV-infected glioma cell lines were measured using MTT assays. (**C**) Cell invasion was inhibited by miR-144-3p in HCMV-infected glioma cells. (**D**) Cell formation was inhibited by miR-144-3p in glioma cells. *: *p* < 0.05; **: *p* < 0.01; ***: *p* < 0.001.

To observe the effects of miR-144-3p in glioma, glioma cell lines were transfected with miR-144-3p mimics, the miR-control, anti-miR-144-3p and the anti-miR-control. qPCR analysis was performed to test the transfection efficiency in glioma cell lines and measure the TOP2A mRNA expression level. miR-144-3p expression was reduced in cells transfected with miR-144-3p mimics compared with other groups, and miR-144-3p reduced the expression of TOP2A (Figure 4A). MTT assays (3-(4,5)-dimethylthiahiazo (-z-y1)-3,5-di-phenytetrazoliumromide) showed that, compared with the control, overexpression of miR-144-3p inhibited cell growth (Figure 4B). As cell invasion is a crucial process in cancer metastasis, we used a cell invasion assay to detect the invasion of glioma cells. High expression of miR-144-3p resulted in a decrease in glioma cell invasion in cells transfected with miR-144-3p compared with the miR-control group (Figure 4C). Moreover, the number of colonies was also decreased in glioma cell lines transfected with miR-144-3p mimics (Figure 4D). These results revealed that accumulated miR-144-3p could restrain cell proliferation, clone formation and migration. Combined with the above data, these results indicate that miR-144-3p directly targets the TOP2A 3′-UTR and inhibits cell growth and migration in glioma cells.

### 2.5. miR-144-3p Suppresses Tumor Growth of U87 Cells In Vivo

**Figure 5:**
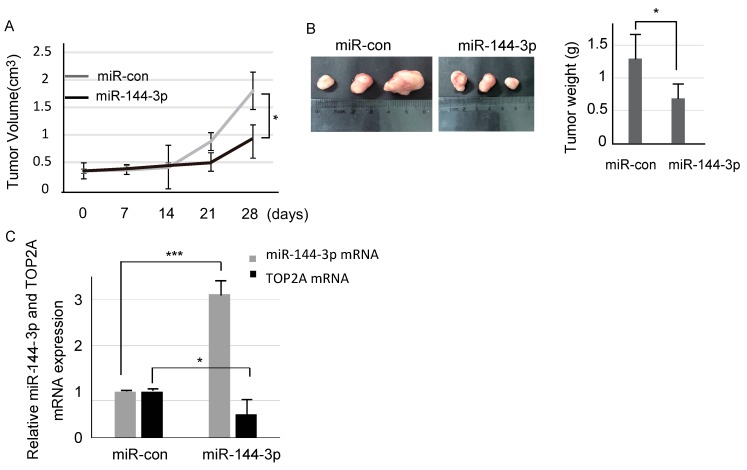
miR-144-3p inhibited tumor growth in vivo. (**A**) Stably transfected HCMV-positive U87 cells were used to establish human tumor xenografts in a mouse model. The diameters of tumors were measured by calipers every seven days, and the tumor volumes were calculated. (**B**) Tumor tissue images are shown, and tumor weights were measured at the end of the experiments. (**C**) miR-144-3p could suppress TOP2A expression in vivo. *: *p* < 0.05; ***: *p* < 0.001.

We evaluated the effects of miR-144-3p on in vivo glioma xenograft growth. U87 cells were transfected with either miR-144-3p or miR-NC. To establish mouse xenograft models, we injected stably transfected cells (1 × 10^6^ cells per condition) into nude mice subcutaneously. The tumors were measured weekly, and miR-144-3p upregulation resulted in tumor growth inhibition in a time-dependent manner (Figure 5A). Hence, compared with the miR-control group, miR-144-3p clearly attenuated tumor growth. After four weeks, the mice were dissected. The comparison of tumor weights indicates that overexpression of miR-144-3p in U87 cells markedly reduces their ability to form tumors (Figure 5B).

Moreover, we detected the relationship between miR-144-3p and TOP2A in mice tumor tissues by Real Time PCR analyses. The results reflected miR-144-3p and TOP2A were negatively correlated (Figure 5C). Taken together, we found accumulated miR-144-3p could suppress TOP2A expression and restrain cell proliferation in vivo.

## 3. Materials and Methods

### 3.1. Clinical Sample Collection

A total of 40 glioma samples, including 29 HCMV-positive tissues and 11 HCMV-negative tissues, were obtained by surgical resection from the Affiliated Hospital of Qingdao University (Qingdao, Shandong, China) between May 2013 and January 2015. The clinical samples were obtained with informed consent, which does not increase the medical costs and pain of the patients. The research materials and research results are used for scientific research purposes, and there is no conflict of interest. The experimental animals involved in this research also approved by the Ethics Committee of the Medical College of Qingdao University. None of the patients received antitumor treatments, such as chemotherapy or radiotherapy, before surgery. The samples were immediately frozen in liquid nitrogen following surgical resection and stored at −80 °C for subsequent analysis.

#### DNA Extraction and Polymerase Chain Reaction

DNeasy Blood & Tissue Kit (69504, QIAGEN, Venlo, The Netherlands) was used for viral DNA extraction from tumor specimens following the manufacturer’s instructions. For PCR, 5 min at 95 °C, followed by 40 cycles of 10 s at 95 °C, 40 s at 60 °C and 30 s at 40 °C. Sequences of primer pairs used in qPCR was the following: IE1 forward, 5′GACTAGTGTGATGCTGGCCAAG-3′ and reverse, 5′-GCTACAATAGCCTCTT CCTCATCTG-3′. 

### 3.2. Immunohistochemical Assays

We performed an immunohistochemical assay for each sample. According to the standard protocol, the major steps included fixing, paraffinizing, dewaxing, dehydrating, antigen retrieval, blocking, incubation with primary antibodies (TOP2A, DAKO, Copenhagen, Denmark), washing, blocking, incubation with secondary antibodies (Santa Cruz Biotechnology, Santa Cruz, CA, USA), antigen–antibody reactions with diaminobenzidine (Maixin, Fuzhou, China) and observations.

### 3.3. Cell line Culture and Viruses

Human glioma cell lines U87 and U251 were obtained from the American Type Culture Collection. The U87 cells were grown in minimum essential medium (HyClone, Logan, UT, USA), and the U251 cells were maintained in Dulbecco’s high-glucose modified Eagle medium (HyClone). All cells were cultured with both 10% FBS (Gibco, GrandIsland, NY, USA) and 1% penicillin/streptavidin (Gibco, USA) and cultured at 37 °C in a humidified atmosphere at 5% CO_2_. HCMV AD169 (France Pasteur Laboratory, Paris, France) was propagated in human embryonic lung fibroblast cells with serum-free medium and stored at −80 °C. HCMV AD169 was tittered by plaque titration and expressed as the number of plaque-forming units (PFU) per milliliter. Glioma cell lines were infected at multiplicity of infection (MOI) of 10. After 12 h, the growth medium was replaced by serum-free medium or indicated medium.

### 3.4. siRNA Transfection

For siRNA transfection, 2 × 10^5^ cells per well were plated in a 6-well plate. After adhering for 24 h, control miRNA (miR-con, UUCUCGAACGUGUCACGUUUU), a miR-144-3p mimic (UCAUGUAGUAGAUAUGACAU) and its corresponding miR-144-3p inhibitor (RiboBio, Guangzhou, China, miR20000436-1-5) were added to the transfection medium for 6 h at 37°C in a CO_2_ incubator. Loss of TOP2A expression was achieved using small interfering RNA (siRNA) of TOP2A (Santa Cruz Biotech, #sc-36695, Santa Cruz, CA, USA). Cell transfection was performed using Lipofectamine 2000 reagent (Invitrogen, Carlsbad, CA, USA) according to the manufacturer’s instructions. The cells were supplemented with normal culture media and cultured at 37 °C with 5% CO_2_ for up to 48 h before harvest.

### 3.5. RNA Extraction and Quantitative RT-PCR Analysis

RNA was isolated from tissues or cells using a mirVana miRNA Isolation Kit (Ambion, Carlsbad, CA, USA) in accordance with the manufacturer's instructions. First-strand cDNA was synthesized using a PrimeScript 1st Strand cDNA Synthesis Kit (Takara, Dalian, China). The extraction concentration was measured with a NanoDrop spectrophotometer (Thermo Fisher, San Jose, CA, USA); the samples were preserved at −80 °C. The cDNA was amplified using Power SYBR Green PCR Master Mix (Applied Biosystems, Carlsbad, CA, USA) with appropriate primers and with an ABI 7500-fast thermocycler (Applied Biosystems, Foster City, CA, USA). The primers used were as follows (forward and reverse, respectively): 5′GCGAGTGTGCTGGTCACTAA′3 and ACAATTGGCCGCTAAACTTG’3 to detect TOP2A mRNA as well as 5′TGCACCACCAACTGCTTAGC′3 and 5’GGCATGGACTGTGGTCATGAG′3 to detect GAPDH. In addition, primers for miR-144-3p and U6 were purchased from Tiangen. U6 and GAPDH served as internal controls, and the relative expression was determined using the 2^−∆∆Ct^ method.

### 3.6. Western Blot Analysis

Snap-frozen tissues were grinded and cells (U87 and U251) in the specific treatments were harvested, and protein supernatants were isolated using cell lysis buffer (Cell Signaling Technology, #9803, Beverly, MA, USA) that was supplemented with phenylmethylsulfonyl fluoride (PMSF, Solarbio, P0100, Beijing, China). The extracted proteins were qualified by the BCA method. The total protein content (30 µg) from the cell lysates was resolved by 10% SDS-PAGE and then transferred to a 0.45-mm nitrocellulose membrane (Millipore, USA-Bedford, MD, USA) for 1 h. The membranes were washed with TBST that contained 5% (*w*/*v*) BSA. The membranes were incubated overnight with primary antibodies for specific HCMV Immediate Early protein (IE1) (ViroStat, #0841, Westbrook, ME, USA), TOP2A (Cell Signaling Technology, #12286, Beverly, MA, USA), and GAPDH (Cell Signaling Technology, #5174, USA). Secondary antibodies were reacted with horseradish peroxidase for 2 h at room temperature. The membranes were then washed three times with TBST at room temperature. Chemiluminescent signals were generated by a Super Signal West Pico Trial Kit (Thermo Fisher, #34094, San Jose, CA, USA) and detected using a Vilber Lourmat imaging system (Vilber Lourmat Corporation, Torcy, France).

### 3.7. MTT Assays

Cell proliferation was determined using MTT assays. The cells were seeded in a 96-well plate with density of the optimized cell number (5000 cells/well). After 24 h of seeding, the cells were treated with siRNA or diluted chemicals at the indicated working concentration. The cells were incubated for the indicated times, and then 20 µL of MTT (5 mg/mL) was added to the wells. Four hours later, the mixed medium was replaced with 150 µL of dimethylsulfoxide (Sigma, St Louis, MO, USA). Next, the 96-well plate was agitated for 15 min at room temperature. The OD value of each well was then measured using a fluorescence microplate reader (Sunrise Remote, Tecan Austria GmbH, Grödig, Austria) at a wavelength of 490 nm.

### 3.8. Luciferase Reporter Assays

The glioma cells were grown to 70–80% confluence in 24-well plates and cotransfected with recombinant plasmids containing the wild-type (wt)/mutant (mut) TOP2A 3′-UTR and miRNA mimics (50 nM) using Lipofectamine™ 2000 reagent (Invitrogen, Carlsbad, CA, USA) according to the manufacturer’s instructions. Luciferase activity was measured 48 h after cotransfection using a Luciferase Assay Kit (Promega, Fitchburg, WI, USA), and the values were normalized against those of *Renilla* luciferase gene activity.

### 3.9. RNA Immunoprecipitation (RIP) Assay

RIP (RNA Immunoprecipitation) assay was performed to analyze the interaction between TOP2A and miR-144-3p using EZ-Magna RIP kit (Millipore, #17-10521, USA). The whole cell extract was incubated with RIP lysis buffer containing magnetic beads conjugated with human anti-Argonaute2 (anti-Ago2) antibody (Abcam, #ab32381, Cambridge, UK) or normal mouse IgG (Millipore, #12-371). IgG was used as a negative control. Then, the samples were incubated with proteinase K for extracting the proteins. Finally, the co-precipitated RNAs were isolated and subjected to qPCR analysis. 

### 3.10. Apoptosis Assays

Apoptotic cells were identified using a terminal deoxynucleotidyl transferase dUTP nick end labeling (TUNEL)-based cell detection kit, POD (Roche Applied Science, Penzberg, Upper Bavaria, Germany). The cells were fixed on slides with 4% paraformaldehyde for 1 h, washed with PBS, and then incubated for 10 min with 3% H_2_O_2_ in methanol at room temperature. After washing with PBS again, the cells were incubated for 2 min on ice with 0.1% Triton X-100 in 0.1% sodium citrate. The slides were then incubated with the TUNEL reaction mixture for 60 min at 37 °C and then rinsed with FBS. The stained slides were examined under light microscopy (Motic B1-223A, Motic Deutschland GmbH, Wetzlar, Germany).

### 3.11. Cell Invasion Assays

Cell invasion ability was examined using Matrigel-coated Transwell chamber inserts with 8 mm pores (Corning Inc., Corning, NY, USA). Stably transduced cells were trypsinized and adjusted to 1 × 10^7^/mL in serum-free MEM/DMEM, and 200 µL of cell suspension (2 × 10^5^ cells) was added to the upper chamber. The lower chamber was filled with 500 µL of 10% FBS-MEM/DMEM. After 72 h, the upper chamber was removed. The cells were then fixed with 70% ethanol and stained with hematoxylin. Afterward, the cells were counted under a light microscope in three randomly selected areas.

### 3.12. Clone-Formation Assays

GBM cells were seeded onto 6 cm dishes at a density of 500 cells/well and cultured for 12 days. The clones were fixed with cold methanol for 15 min and then stained with 0.1% crystal violet for 10 min. The clone-formation ability was evaluated as the number of colonies.

### 3.13. Xenograft Experiments

All mice were obtained from the Institute of Laboratory Animal Science and maintained specific pathogen-free conditions in accordance with the approved guidelines for animal welfare in China. The mice were divided into the following two groups: a miR-con group and a miR-144-3p group. A total of 3 × 10^6^ cells were subcutaneously injected into the right armpits of 4-week-old BALB/c mice. During this experiment, the mice were weighed by a table balance, and tumors were measured with calipers every week. After 5 weeks, the mice were euthanized using a subcutaneous injection of sodium pentobarbital (50 mg/kg), after which the tumor tissue volume was measured. The tumor volume was calculated by the following formula: V (mm^3^) = 0.5 × length × (width)^2^.

### 3.14. Software Support and Statistical Analysis

All images were formatted for optimal presentation using Adobe Illustrator CS4 (Adobe Systems, San Jose, CA, USA). The intensity of IHC images were determined using the IHC Toolbox plugin for ImageJ (NIH, Bethesda, MD, USA) (https://imagej.nih.gov/ij/). In this study, we defined the relative TOP2A expression > 7 as high expression and 7 is derived from the mean values of all samples. To determine statistical significance between two groups, a Student’s t-test was performed to calculate the associated *p*-values. Statistical significance between multiple groups was evaluated by one way analysis of variance (ANOVA) followed by a Newman-Keuls post hoc test using GraphPad Prism 5 (GraphPad Software, Inc., La Jolla, CA, USA). 

## 4. Conclusions

GBM, the most prevalent and aggressive tumor, is a primary malignant tumor with a poor prognosis and high morbidity [1]. Current treatment lacks efficacy, indicating that new therapeutic approaches are needed. Accumulating evidence indicates that HCMV is related to GBM and can modulate glioma stem biology processes, including cell proliferation, differentiation and metastasis [10]. Previous studies have revealed that proteins IE1 and IE2 are highly expressed in GBM and increase TOP2A expression [27,28]. TOP2A is highly expressed in some types of cancers, including breast cancer, hepatocellular carcinoma, and glioma, and serve as a biomarker for these cancers [29,30]. TOP2A plays a vital role in both cancer-inhibiting and cancer-promoting processes [31]. The tissue type and the tumor microenvironment have different effects on the process of carcinogenesis due to the different roles of TOP2A. In our study, there was a majority of HCMV-positive tumor tissues expressing high levels of TOP2A mRNA compared to HCMV-negative control tissues. Moreover, we found both high TOP2A mRNA expression and protein expression in HCMV-infected glioma cells. High TOP2A expression in GBM was correlated with cell proliferation and malignant transformation in HCMV-positive glioma cells. However, the concrete mechanism and therapeutic targets of GBM have not yet been fully elucidated.

miRNAs regulate a variety of biological processes and functions involved in physiological and pathological conditions, including cell proliferation, cell apoptosis, development, and metabolism [32,33]. Various studies have reported that miRNAs are associated with tumors [34]. To date, the function of miR-144-3p in glioma is unclear. Here, we found that miR-144-3p had a lower expression level in HCMV-positive glioma tissues and HCMV-infected glioma cell lines than in control tissues. Moreover, upregulation of miR-144-3p expression markedly inhibited glioma cell viability and invasion. To better understand the mechanism of miR-144-3p/TOP2A affected cell growth and invasive ability of HCMV-infected glioma cells, the apoptosis and metastasis-related proteins (Caspase-3, Bcl-2, Bax, Apaf-1, MMP, TIMP, Geletinase, Calpain, Cathepsin and etc.) should be detected in the further study. Taken together, these results show that miR-144-3p expression gradually decreased with increased glioma malignancy. We identified TOP2A as a direct target gene of miR-144-3p for HCMV-infected glioma cells. Forced expression of miR-144-3p was explored to lower the expression levels of TOP2A in glioma cells, which promoted malignant progression. This finding suggested that miR-144-3p attenuated the viability and invasion of glioma cells by targeting TOP2A. Additionally, we verified that, compared with the control mice, mice injected with stably transduced miR-144-3p glioma cells had fewer metastases in vivo. These observations indicate that miR-144-3p induces cell apoptosis and inhibits cell migration by targeting TOP2A, thereby inhibiting glioma cell growth.

## Figures and Tables

**Table 1:** Correlations between TOP2A expression in glioma and clinical characteristics.

Group	No.	Relative TOP2A Expression		*p* Value
		Low	High	
Gender				
Male	15	7	8	0.324
Female	25	17	8	
Age				
≤50	28	16	12	0.845
≥50	12	8	4	
WHO grade				
I-II stage	24	22	2	0.009 *
III-IV stage	16	2	14	
HCMV infection				
HCMV-positive	29	13	16	0.012 *
HCMV-negative	11	11	0	
KPS				
≤80	8	4	4	0.798
≥80	32	19	13	

WHO, World Health Organization; KPS, Karnofsky performance status. * *p* < 0.05.

## Supplementary Materials

The following are available online. Figure S1: Relative expression levels of the IE1 were measured by PCR (A) and western blot (B) in glioblastoma tissues; Figure S2: The protein expression level of IE1 was measured by immunohistochemistry in glioblastoma tissues (40×); Figure S3: The protein expression level of TOP2A was measured by immunohistochemistry in glioblastoma tissues (40×); Figure S4: The miR-144-3p and TOP2A were oppositely affected by HCMV infection with anti-miR-144-3p (A) and miR-144-3p (B) treatment; Table S1: Correlations between TOP2A expression in glioma and clinical characteristics.
