# Supplementary material for: The Inhibition of miR-144-3p on Cell Proliferation and Metastasis by Targeting TOP2A in HCMV-Positive Glioblastoma Cells
Source: Molecules. 2018 Dec 10;23(12):3259. doi: 10.3390/molecules23123259 (PMC6320803; doi:10.3390/molecules23123259)
Supplement: Supplementary file 1 [file molecules-23-03259-s001.zip › molecules-397639-revised supplementary/Figure S1.pdf]

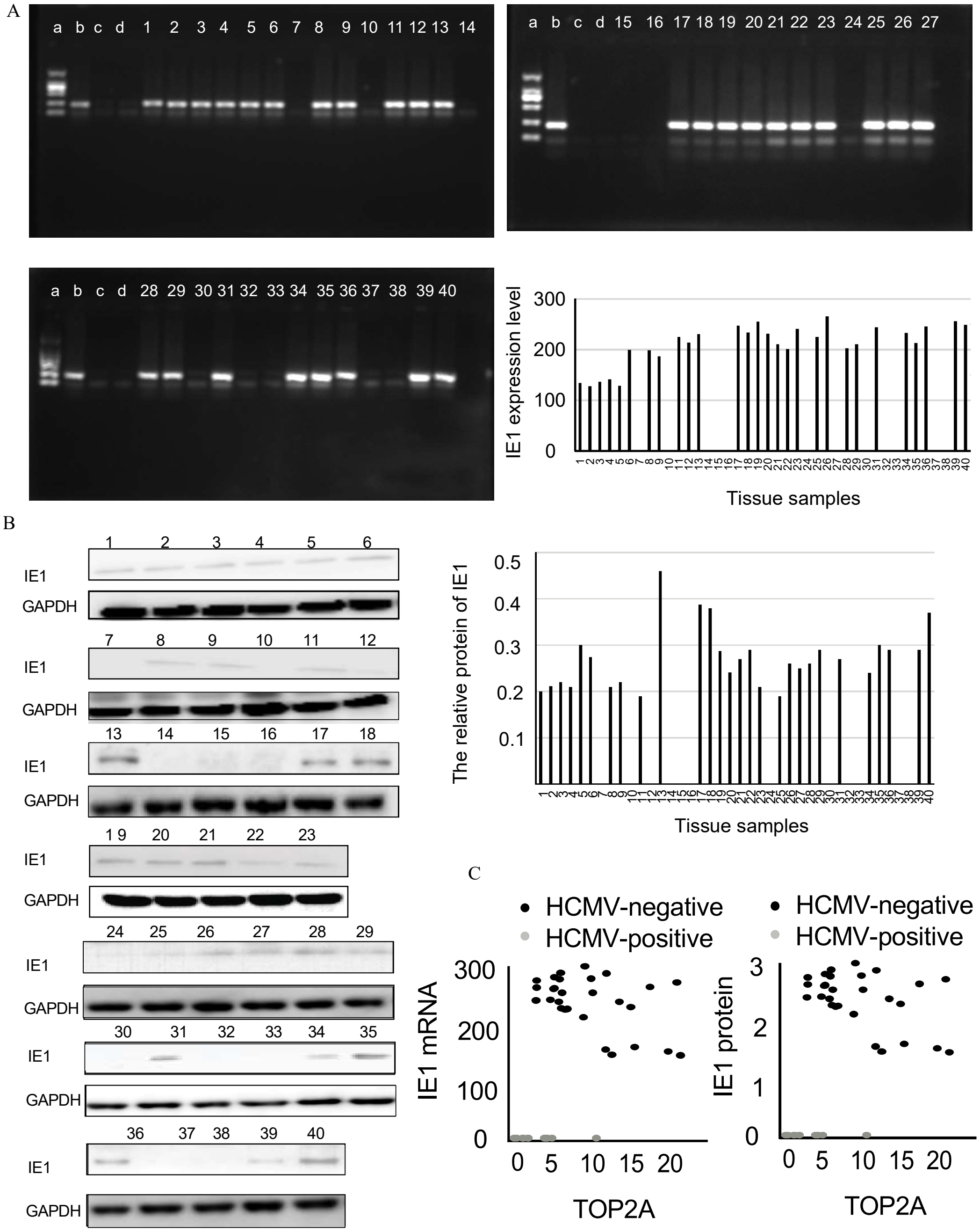

Figure S1A. Relative expression levels of the IE1 were measured by PCR (A) and western blot (B) in glioblastoma tissues. a. Marker b. positive control (HCMV-infected human lung fibroblasts) c. negative control (HCMV-uninfected human lung fibroblasts) d. water #1-#40. glioblastoma samples. The sequence number is consistent with the raw data in Table S1. (C) The relationship between IE1 and TOP2A expression was investigated.
