# Supplementary material for: The Inhibition of miR-144-3p on Cell Proliferation and Metastasis by Targeting TOP2A in HCMV-Positive Glioblastoma Cells
Source: Molecules. 2018 Dec 10;23(12):3259. doi: 10.3390/molecules23123259 (PMC6320803; doi:10.3390/molecules23123259)
Supplement: Supplementary file 1 [file molecules-23-03259-s001.zip › molecules-397639-revised supplementary/Figure S2.pdf]

A

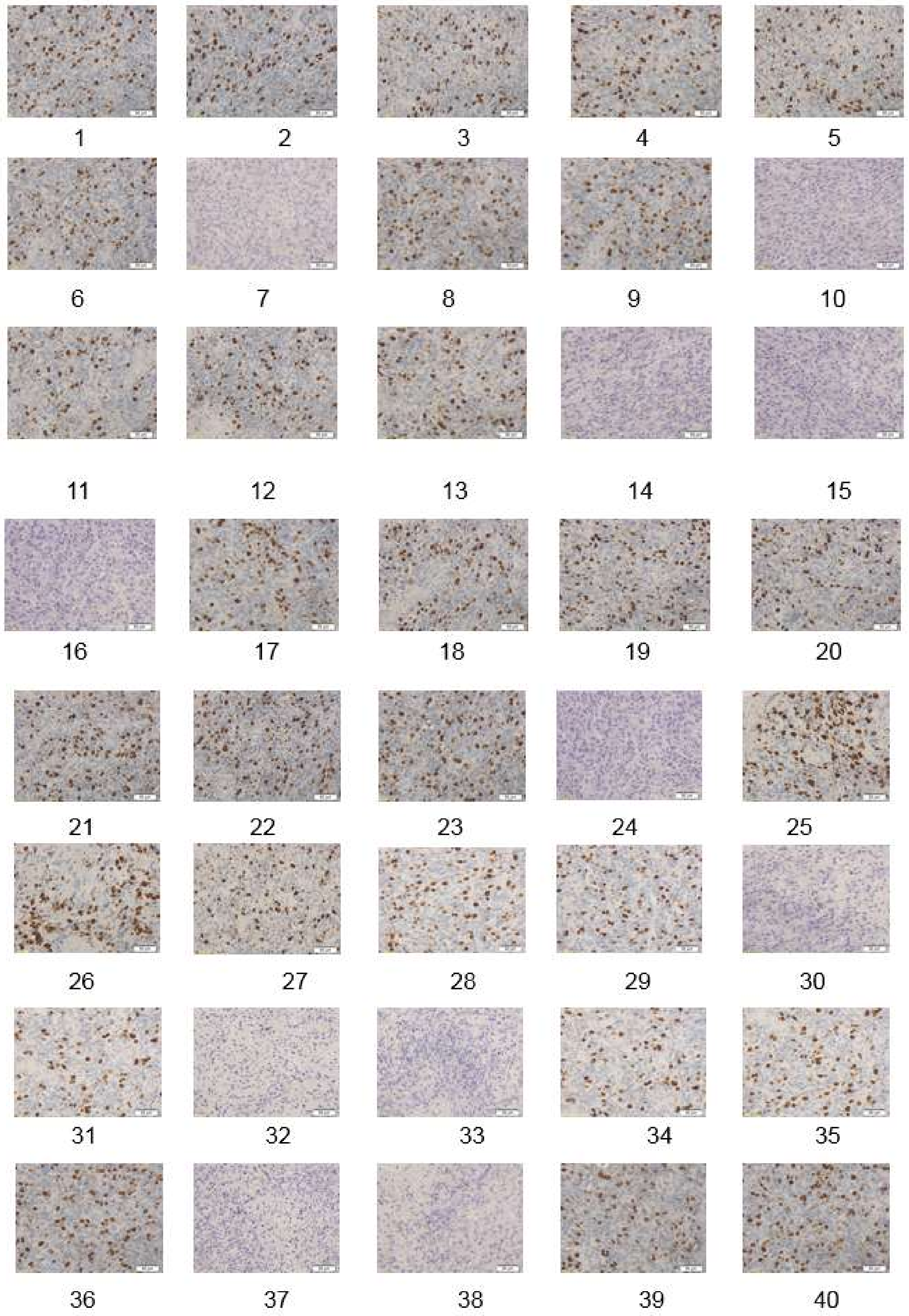
1
2
3
4
5
6
7
8
9
10
11
12
13
14
15
16
17
18
19
20
21
22
23
24
25
26
27
28
29
30
31
32
33
34
35
36
37
38
39
40

B

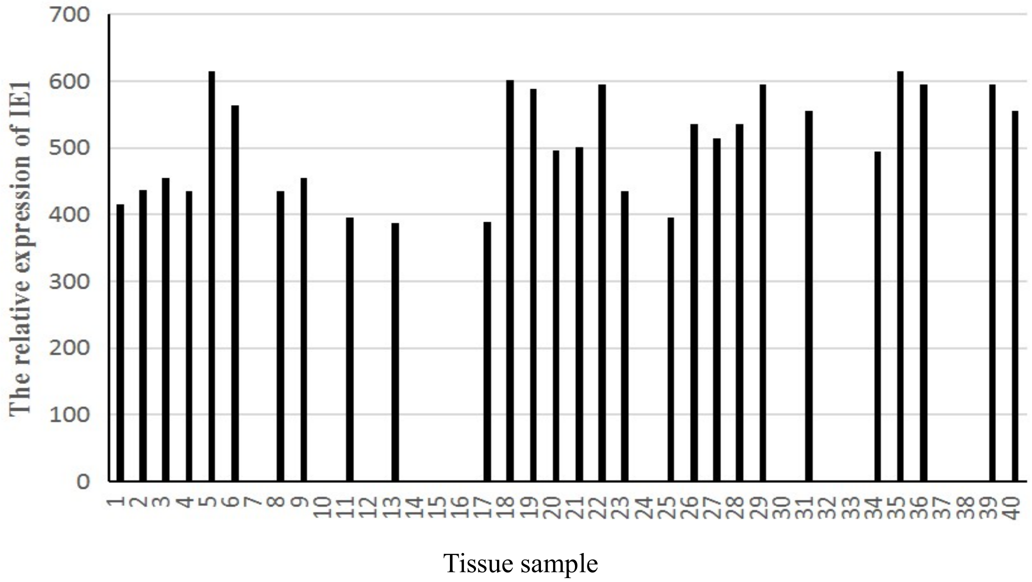

Figure S2. A. The protein expression level of IE1 was measured by immunohistochemistry in glioblastoma tissues (40x). #1-#40. glioblastoma samples. The sequence number is consistent with the raw data in Table S1. B. The intensity of IHC images were determined using the IHC Toolbox plugin for ImageJ.
