# Supplementary material for: The Inhibition of miR-144-3p on Cell Proliferation and Metastasis by Targeting TOP2A in HCMV-Positive Glioblastoma Cells
Source: Molecules. 2018 Dec 10;23(12):3259. doi: 10.3390/molecules23123259 (PMC6320803; doi:10.3390/molecules23123259)
Supplement: Supplementary file 1 [file molecules-23-03259-s001.zip › molecules-397639-revised supplementary/Figure S4.pdf]

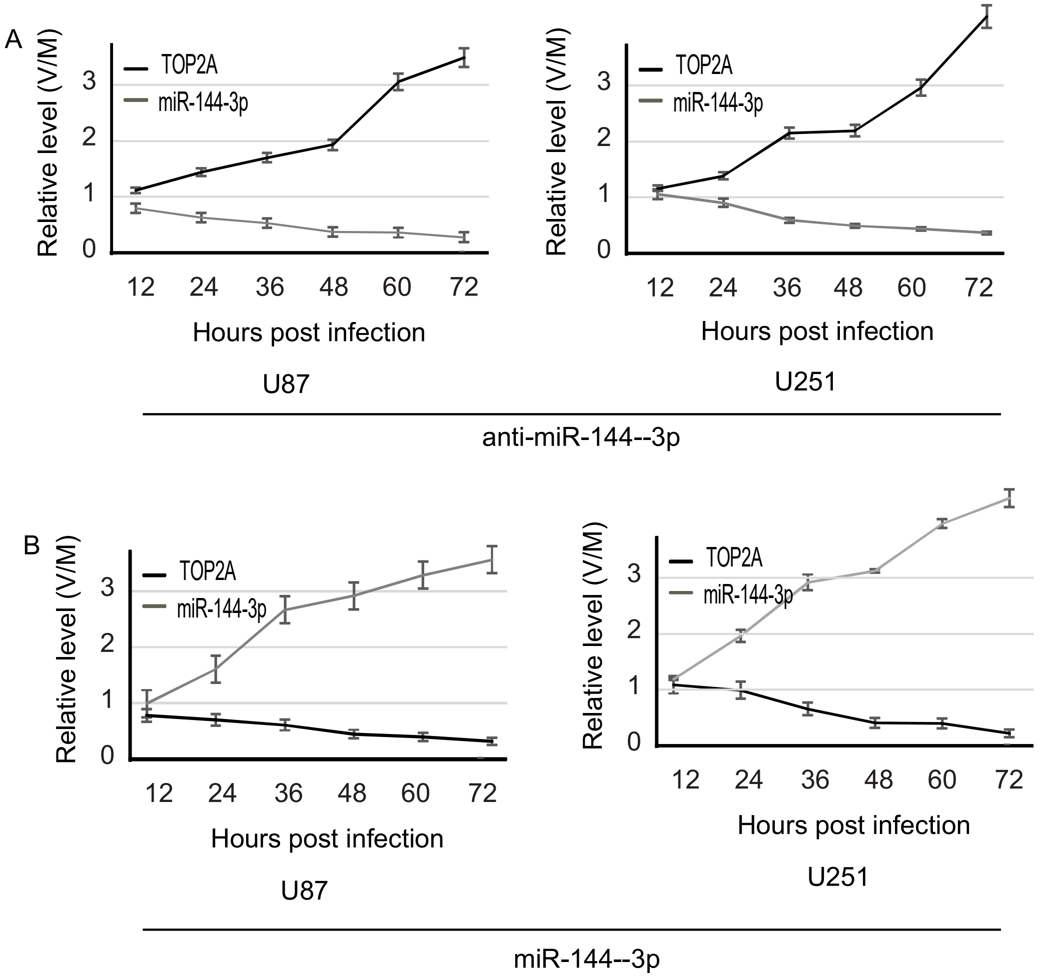

Figure S4. The miR-144-3p and TOP2A were oppositely affected by HCMV infection with anti-miR-144--3p (A) and miR-144-3p (B) treatment.
