# Supplementary material for: The Inhibition of miR-144-3p on Cell Proliferation and Metastasis by Targeting TOP2A in HCMV-Positive Glioblastoma Cells
Source: Molecules. 2018 Dec 10;23(12):3259. doi: 10.3390/molecules23123259 (PMC6320803; doi:10.3390/molecules23123259)
Supplement: Supplementary file 1 [file molecules-23-03259-s001.zip › molecules-397639-revised supplementary/Table S1.pdf]

TableS1：Correlations between TOP2A expression in glioma and clinical characteristics.

NO.	Sexy	Age	shape of tumor	WHO	Metastatic	IE1	IE1 (+/-)	TOP2A	miR-144-3p
1	M	47	2.9*1.9*1.1cm	Glioblastoma multiforme (IV)	N	1.34	+	20.39	0.599
2	F	50	2.5*2*1cm	Glioblastoma multiforme (IV)	Y	1.28	+	22	0.934
3	F	20	3.5*3*1cm	Anaplastic Glioma (III)	N	1.37	+	12.2	2.011
4	M	61	2*2*0.5cm	Glioblastoma multiforme (IV)	Y	1.41	+	16	2.015
5	F	63	3.3*2*2cm	Glioblastoma multiforme (IV)	N	1.29	+	13	2.978
6	F	42	3*2.2*0.9cm	Anaplastic Glioma (III)	N	2	+	7.2	3.478
7	M	39	2*1.2*1cm	Astrocytoma (II)	N	0	-	4.32	11.757
8	M	53	3.5*1.5*1.3cm	Oligodendroglioma (I-II)	N	1.99	+	6.9	4.775
9	F	44	3.8*2*1cm	Astrocytoma (II-III)	N	1.87	+	9.3	3.789
10	F	8	2cm in diameter	Astrocytoma (I)	N	0	-	0.23	23.244
11	F	47	3cm in diameter	Astrocytoma (II)	N	2.25	+	6.5	4.779
12	F	11	2cm in diameter	Low grade Glioma (I)	N	2.14	+	5	5.773666667
13	F	18	2*1.6cm	Astrocytoma (II)	N	2.31	+	5.4	5.304
14	F	48	3.9*3.5*1cm	Oligodendroglioma (II-III)	N	0	-	4.5	10.0024
15	M	37	3*2*1cm	Low grade Glioma (I)	N	0	-	4.21	10.9987
16	F	8	2*2*1.9cm	Astrocytoma (I)	N	0	-	1.48	13.5322
17	M	46	3.1*3*1.1cm	Anaplastic Glioma (III)	N	2.47	+	10.32	3.957
18	F	26	4*3.1*3cm	Anaplastic Glioma (III)	N	2.34	+	18	1.978
19	M	32	3.9*3.8*3cm	Glioblastoma multiforme (IV)	Y	2.55	+	12.3	2.133
20	M	38	2*1.6*1.6cm	Astrocytoma (II)	N	2.32	+	5.5	5.298
21	F	25	4.2*3.3*2.9cm	Glioblastoma multiforme (IV)	Y	2.11	+	14	2.0197
22	M	46	5.1*3.1*2cm	Glioblastoma multiforme, (IV)	Y	2.01	+	6.4	5.0123
23	M	28	4.4*2.1*2.1cm	Glioblastoma multiforme (IV)	N	2.41	+	21.5	1.995
24	M	58	2.9*2.5*2.1cm	Anaplastic Glioma (III)	N	0	-	5.2	3.8001
25	M	49	3*2.9*2.5cm	Astrocytoma (II-III)	N	2.25	+	10.5	1.94133333
26	M	65	1.5cm in diameter	Anaplastic Glioma (III)	N	2.66	+	9.5	2.838
27	M	43	3*2.8*0.8cm	Anaplastic Glioma (III)	N	0	+	11	2.564
28	F	40	4*2.9*2.1cm	Glioblastoma multiforme (IV)	Y	2.03	+	15.5	1.291
29	M	51	2*1.3*1cm	Astrocytoma (II)	N	2.11	+	6.1	4.256
30	F	59	1.2*3cm	Astrocytoma (II)	N	0	-	2.1	12.4536
31	F	26	2.1*1.7cm	Astrocytoma (I)	N	2.44	+	3.2	7.551

32	F	26	2.3*2.1*2cm	Astrocytoma (I)	N	0	-	2.05	8.0737
33	F	77	3.5*3.2*2.1cm	Astrocytoma (I)	N	0	-	4.6	6.9689
34	F	49	1.9*1.6*0.9cm	Astrocytoma (II)	N	2.33	+	3.2	5.96133333
35	F	51	2.2*1.8*0.9cm	Low grade Glioma (I-II)	N	2.13	+	3.1	5.91566667
36	F	39	2.3*1.1*0.8cm	Oligodendroglioma (II)	N	2.46	+	6.2	4.1245
37	F	19	1cm in diameter	Astrocytoma (I)	N	0	-	1.32	17.0122
38	F	68	2.7*2.5*1.5cm	Astrocytoma (II)	N	0	-	0.58	22.9292
39	F	81	1.6*1.0*0.8cm	Astrocytoma (II)	N	2.56	+	6.3	3.53666667
40	F	26	3.1*1.9*1.5cm	Astrocytoma (II)	N	2.49	+	5.5	5.45933333

Sexy: Male (M)/ Female (F), Metastatic status: with metastases(Y) /without metastases (N), IE1: the relative expression of IE1 and positive (+)/ negative (-), TOP2A: the relative expression of TOP2A, miR-144-3p: the relative expression of miR-144-3p.
